# Supplementary material for: Detection of Lymphoid Markers (CD3 and PAX5) for Immunophenotyping in Dogs and Cats: Comparison of Stained Cytology Slides and Matched Cell Blocks
Source: Vet Sci. 2023 Feb 15;10(2):157. doi: 10.3390/vetsci10020157 (PMC9963973; doi:10.3390/vetsci10020157)
Supplement: Supplementary file 1 [file vetsci-10-00157-s001.zip › Figure S1 supplemental with legend_revised.pdf]

Figure S1. Examples of background and non-specific staining in immunolabeling for CD3 and PAX5 on previous-stained cytology slides and on cell blocks sections.

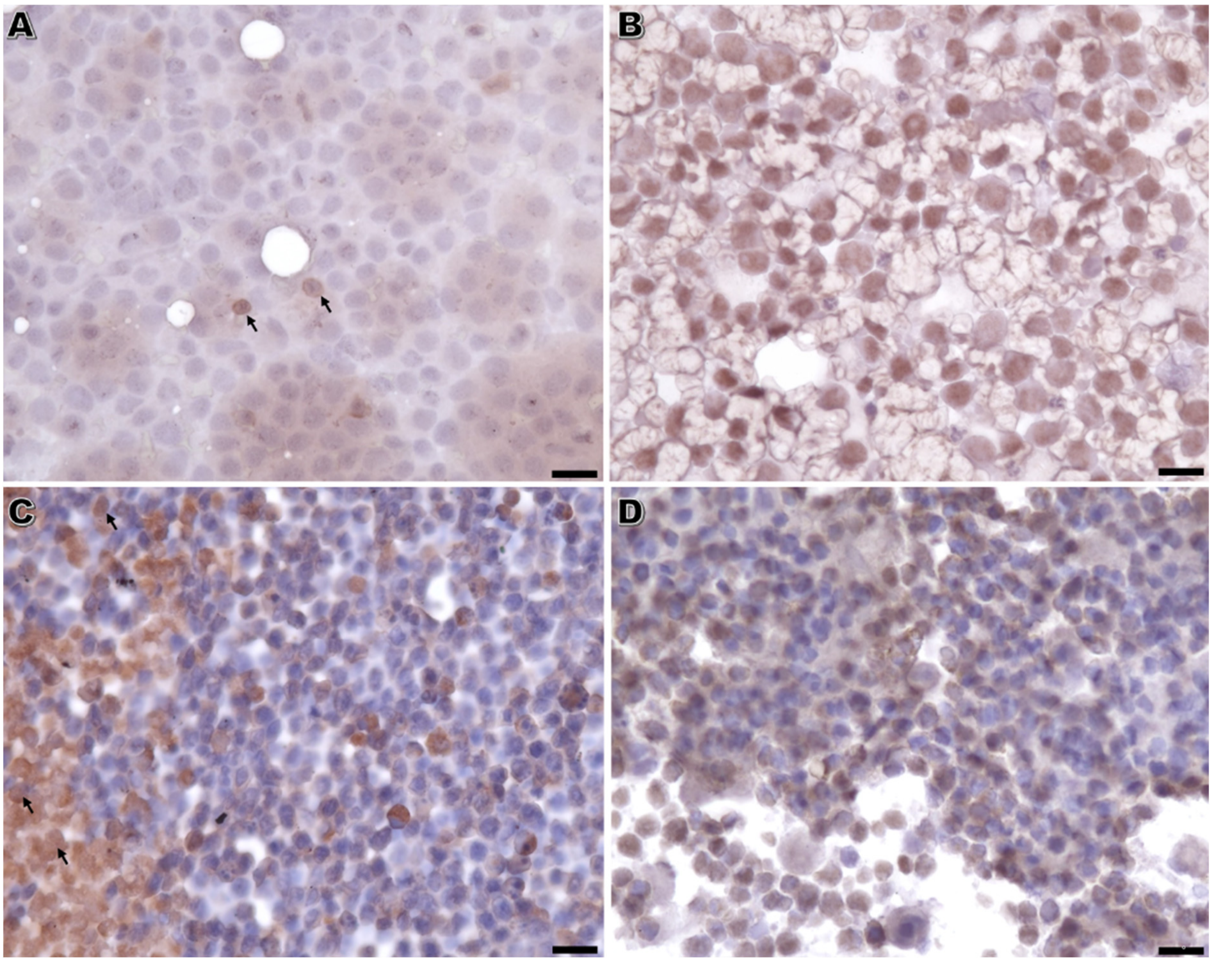

A) CD3 immunolabeling in a nodal fine-needle aspiration (FNA) slide (non-coverslipping, 16 months of archive) of a B-cell lymphoma of a dog. Note the moderate non-cellular background staining (positive membranous staining was observed in less than 25% of the cells - *arrows*). **B)** PAX5 immunostaining in a nodal FNA slide (non-coverslipping and 40 months of archive) of a dog with a B-cell lymphoma. Slight background staining was noted. The nuclear staining in more than 75% of cells is associated with a diffuse non-specific cytoplasmic weak staining. **C)** Cell block section immunolabelled with anti-CD3 of a pleural effusion of a dog. Non-specific staining is observed in cellular structures (*arrows*). Phenotype could not be assigned in this case **D)** PAX5 immunoreaction in a cell block section of a FNA needle rinse of a lymph node in a cat. Background and non-specific staining cytoplasmic staining (nuclear staining is the expected pattern in PAX5) are present. Diaminobenzidine chromogen, Haematoxylin counterstain. bar = 24 µm (A and B) and 14 µm (C and D).
