# Supplementary material for: Detection of Lymphoid Markers (CD3 and PAX5) for Immunophenotyping in Dogs and Cats: Comparison of Stained Cytology Slides and Matched Cell Blocks
Source: Vet Sci. 2023 Feb 15;10(2):157. doi: 10.3390/vetsci10020157 (PMC9963973; doi:10.3390/vetsci10020157)

Figure S2: Negative and positive controls of the immunostaining protocol. **A)** Cytology smear used as negative control. No staining inn cellular or non-cellular structures was observed, even in the presence of a bloody background. **B)** Histologic section of a normal lymph node collected during necropsy used as negative control. Similarly, no staining was noted and the background was completely clear. **C)** Membranous CD3 staining in normal lymphocytes from the paracortical areas of a hyperplastic lymph node used as positive control. **D)** Clear and strong nuclear PAX5 immunolabeling in the cortical B-cell lymphocytes of a hyperplastic lymph node, used as the histologic positive control. Diaminobenzidine chromogen, Haematoxylin counterstain. bar = 40 µm (A) and 67 µm (B, C and D).

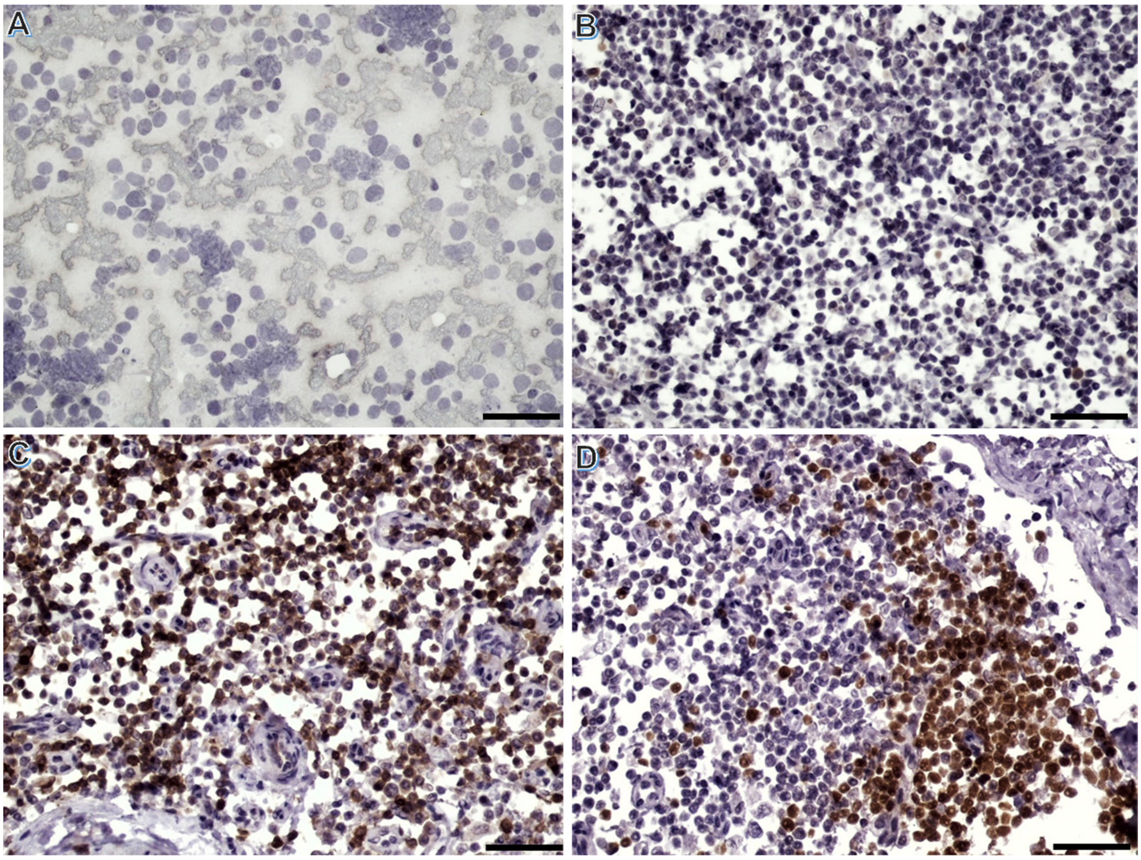

Supplement: Supplementary file 1 [file vetsci-10-00157-s001.zip › Figure S2 supplemental with legend_new.pdf]
