# Supplementary material for: Detection of Lymphoid Markers (CD3 and PAX5) for Immunophenotyping in Dogs and Cats: Comparison of Stained Cytology Slides and Matched Cell Blocks
Source: Vet Sci. 2023 Feb 15;10(2):157. doi: 10.3390/vetsci10020157 (PMC9963973; doi:10.3390/vetsci10020157)
Supplement: Supplementary file 1 [file vetsci-10-00157-s001.zip › Table S2 supplemental_revised.pdf]

Supplemental material

Sampaio et al. Detection of lymphoid markers (CD3 and PAX5) for immunophenotyping in dogs and cats: comparison of stained cytology slides and matched cell blocks

Table S2: Raw data including the type of specimen, pre-analytical variables and results of the immunolabeling for PAX5 in previous Romanowsky-stained cytology slides (RSC) and cell blocks (CB) section

					RSC					CB				
**Case**	**Specimen**	**Species**	**Coverslipping**	**Archive Time (Categories)**	**Positivity (Observer1)**	**Positivity (Observer2)**	**Intensity**	**Background**	**Non-Specific Stain**	**Positivity (Observer1)**	**Positivity (Observer2)**	**Intensity**	**Background**	**Non-Specific Stain**
**1**	Needle rinse (lymph node)	Dog	Yes	1	1	1	2	0	0	1	1	3	1	0
**2**	Needle rinse (lymph node)	Dog	Yes	1	1	1	3	0	0	2	1	3	0	0
**3**	Needle rinse (lymph node)	Cat	No	1	4	4	3	2	1	4	3	3	2	0
**4**	Effusion Cell Block	Cat	Yes	1	2	1	2	0	0	1	1	3	1	0
**5**	Needle rinse (intestinal mass)	Cat	Yes	1	1	1	1	0	0	1	1	3	0	0
**6**	Needle rinse (lymph node)	Dog	Yes	2	4	4	3	0	0	4	4	3	1	0
**7**	Needle rinse (lymph node)	Dog	No	2	3	3	2	0	0	4	4	3	0	1
**8**	Effusion Cell Block	Cat	No	2	1	1	3	0	0	1	0	3	0	0
**9**	Needle rinse (lymph node)	Cat	No	2	1	0	3	0	0	0	0	0	0	0
**10**	Effusion Cell Block	Cat	No	2	1	1	2	0	1	0	0	0	0	1
**11**	Needle rinse (spleen)	Dog	Yes	2	4	4	3	0	1	2	2	3	0	0
**12**	Needle rinse (lymph node)	Cat	Yes	2	1	0	3	0	0	1	0	3	0	0
**13**	Effusion Cell Block	Cat	Yes	2	1	0	2	0	0	1	1	3	0	0
**14**	Needle rinse (lymph node)	Dog	Yes	2	4	4	2	0	0	4	4	3	0	0
**15**	Effusion Cell Block	Cat	Yes	2	1	1	2	0	0	1	1	3	0	0

Legend

Archive time: **1** ≤ 15 days; **2** >15 days ≤5 months; **3** >5 ≤12 months; **4** >12 ≤24 months; **5** >24 ≤60 months; **6** >60 months

Positivity: **0** no positive cell; score **1** ≤25%; score **2** >25 and ≤50%; score **3** >50 and ≤75%; score **4** > 75% of positive cells

Intensity: **0** no positive cell; level **1** weak; level **2** moderate; level **3** intense staining, as compared to controls

Background: **0**: no background staining; **1** weak; **2** moderate; **3** intense background staining

Non-specific staining: **0**: absent; **1**: present.

NA: cells not-available/loss of material during the protocol

Supplemental material

Sampaio et al. Detection of lymphoid markers (CD3 and PAX5) for immunophenotyping in dogs and cats: comparison of stained cytology slides and matched cell blocks

					ICC Cytology					IHC Cell block				
**Case**	**Specimen**	**Species**	**Coverslipping**	**Archive Time (Categories)**	**Positivity (Observer1)**	**Positivity (Observer2)**	**Intensity**	**Background**	**Non-Specific Stain**	**Positivity (Observer1)**	**Positivity (Observer2)**	**Intensity**	**Background**	**Non-Specific Stain**
**16**	Effusion Cell Block	Cat	Yes	2	1	1	3	0	0	1	1	3	0	0
**17**	Needle rinse (lymph node)	Dog	Yes	3	4	4	2	2	0	4	4	3	2	0
**18**	Needle rinse (lymph node)	Cat	Yes	3	0	0	0	2	1	4	4	3	1	0
**19**	Needle rinse (lymph node)	Dog	Yes	3	1	1	2	0	0	1	1	3	0	0
**20**	Needle rinse (lymph node)	Dog	Yes	3	4	4	3	2	0	4	4	3	0	0
**21**	Needle rinse (lymph node)	Dog	Yes	3	4	4	2	0	0	4	4	3	1	0
**22**	Needle rinse (lymph node)	Dog	Yes	3	3	3	2	0	1	4	3	3	1	0
**23**	Needle rinse (lymph node)	Dog	Yes	3	4	4	3	0	0	4	4	3	0	0
**24**	Needle rinse (lymph node)	Dog	Yes	3	4	4	3	1	0	3	3	1	0	0
**25**	Effusion Cell Block	Cat	Yes	4	1	1	3	1	0	1	1	3	0	0
**26**	Effusion Cell Block	Dog	Yes	4	1	0	3	0	0	1	0	3	0	0
**27**	Effusion Cell Block	Dog	Yes	4	1	0	1	2	0	0	0	0	0	0
**28**	Needle rinse (lymph node)	Dog	Yes	4	0	0	0	0	0	0	0	0	0	0
**29**	Needle rinse (lymph node)	Dog	Yes	4	1	1	2	0	0	1	1	3	0	0
**30**	Effusion Cell Block	Dog	No	4	1	1	2	0	0	0	0	0	0	1

Legend

Archive time: **1** ≤ 15 days; **2** >15 days ≤5 months; **3** >5 ≤12 months; **4** >12 ≤24 months; **5** >24 ≤60 months; **6** >60 months

Positivity: **0** no positive cell; score **1** ≤25%; score **2** >25 and ≤50%; score **3** >50 and ≤75%; score **4** > 75% of positive cells

Intensity: **0** no positive cell; level **1** weak; level **2** moderate; level **3** intense staining, as compared to controls

Background: **0**: no background staining; **1** weak; **2** moderate; **3** intense background staining

Non-specific staining: **0**: absent; **1**: present.

NA: cells not-available/loss of material during the protocol

Supplemental material

Sampaio et al. Detection of lymphoid markers (CD3 and PAX5) for immunophenotyping in dogs and cats: comparison of stained cytology slides and matched cell blocks

					ICC Cytology					IHC Cell block				
**Case**	**Specimen**	**Species**	**Coverslipping**	**Archive Time (Categories)**	**Positivity (Observer1)**	**Positivity (Observer2)**	**Intensity**	**Background**	**Non-Specific Stain**	**Positivity (Observer1)**	**Positivity (Observer2)**	**Intensity**	**Background**	**Non-Specific Stain**
**31**	Needle rinse (lymph node)	Dog	No	4	4	4	1	1	0	4	4	3	0	1
**32**	Needle rinse (lymph node)	Dog	No	4	4	4	1	2	0	4	4	2	1	0
**33**	Needle rinse (lymph node)	Dog	No	4	1	1	2	0	0	1	0	3	0	0
**34**	Needle rinse (cutaneous mass)	Dog	No	4	NA	NA	NA	NA	NA	0	0	0	0	0
**35**	Effusion Cell Block	Dog	Yes	5	0	0	0	1	0	1	1	3	0	0
**36**	Effusion Cell Block	Cat	Yes	5	2	1	2	0	0	2	2	3	0	0
**37**	Needle rinse (lymph node)	Dog	No	5	4	4	1	0	1	4	4	3	0	1
**38**	Needle rinse (lymph node)	Dog	Yes	5	0	0	0	1	0	0	0	0	0	1
**39**	Needle rinse (lymph node)	Dog	No	5	1	0	1	1	1	1	1	3	0	0
**40**	Needle rinse (lymph node)	Dog	No	5	4	4	2	0	0	3	3	3	0	1
**41**	Needle rinse (lymph node)	Dog	No	5	4	3	1	1	1	4	2	2	0	1
**42**	Needle rinse (lymph node)	Dog	No	5	0	0	0	1	1	3	0	2	0	1
**43**	Needle rinse (lymph node)	Dog	No	5	4	4	2	1	0	2	0	3	2	0
**44**	Effusion Cell Block	Cat	No	5	NA	0	NA	NA	NA	NA	1	NA	NA	NA
**45**	Effusion Cell Block	Cat	No	5	1	1	2	1	0	1	1	2	0	0

Legend

Archive time: **1** ≤ 15 days; **2** >15 days ≤5 months; **3** >5 ≤12 months; **4** >12 ≤24 months; **5** >24 ≤60 months; **6** >60 months

Positivity: **0** no positive cell; score **1** ≤25%; score **2** >25 and ≤50%; score **3** >50 and ≤75%; score **4** > 75% of positive cells

Intensity: **0** no positive cell; level **1** weak; level **2** moderate; level **3** intense staining, as compared to controls

Background: **0**: no background staining; **1** weak; **2** moderate; **3** intense background staining

Non-specific staining: **0**: absent; **1**: present.

NA: cells not-available/loss of material during the protocol

Supplemental material

Sampaio et al. Detection of lymphoid markers (CD3 and PAX5) for immunophenotyping in dogs and cats: comparison of stained cytology slides and matched cell blocks

					ICC Cytology					IHC Cell block				
**Case**	**Specimen**	**Species**	**Coverslipping**	**Archive Time (Categories)**	**Positivity (Observer1)**	**Positivity (Observer2)**	**Intensity**	**Background**	**Non-Specific Stain**	**Positivity (Observer1)**	**Positivity (Observer2)**	**Intensity**	**Background**	**Non-Specific Stain**
**46**	Effusion Cell Block	Cat	No	5	0	0	0	0	0	1	0	3	0	0
**47**	Effusion Cell Block	Cat	Yes	6	3	1	2	0	0	4	4	3	1	0
**48**	Effusion Cell Block	Cat	Yes	6	0	0	0	0	0	1	0	3	0	0
**49**	Effusion Cell Block	Cat	Yes	6	NA	0	NA	1	0	2	1	3	0	0
**50**	Effusion Cell Block	Cat	Yes	6	3	3	2	0	0	2	1	2	0	1
**51**	Effusion Cell Block	Cat	Yes	6	0	0	0	0	1	1	1	3	0	0
**52**	Effusion Cell Block	Cat	Yes	6	0	0	0	0	0	1	0	2	0	0
**53**	Effusion Cell Block	Cat	Yes	6	1	1	2	0	0	1	1	3	0	0
**54**	Effusion Cell Block	Cat	No	6	0	0	0	1	0	1	1	2	0	0
**55**	Effusion Cell Block	Cat	No	6	1	1	2	0	0	1	1	3	0	0
**56**	Effusion Cell Block	Cat	No	6	1	1	1	0	0	1	1	3	0	0
**57**	Effusion Cell Block	Cat	No	6	2	0	1	2	1	4	4	3	1	0

Legend

Archive time: **1** ≤ 15 days; **2** >15 days ≤5 months; **3** >5 ≤12 months; **4** >12 ≤24 months; **5** >24 ≤60 months; **6** >60 months

Positivity: **0** no positive cell; score **1** ≤25%; score **2** >25 and ≤50%; score **3** >50 and ≤75%; score **4** > 75% of positive cells

Intensity: **0** no positive cell; level **1** weak; level **2** moderate; level **3** intense staining, as compared to controls

Background: **0**: no background staining; **1** weak; **2** moderate; **3** intense background staining

Non-specific staining: **0**: absent; **1**: present.

NA: cells not-available/loss of material during the protocol
