# Supplementary material for: Detection of Lymphoid Markers (CD3 and PAX5) for Immunophenotyping in Dogs and Cats: Comparison of Stained Cytology Slides and Matched Cell Blocks
Source: Vet Sci. 2023 Feb 15;10(2):157. doi: 10.3390/vetsci10020157 (PMC9963973; doi:10.3390/vetsci10020157)
Supplement: Supplementary file 1 [file vetsci-10-00157-s001.zip › Tables S3-S10 supplemental_revised.pdf]

Tables S3–S6: Contingency tables comparing the results of immunolabeling for CD3 applied to previous Romanowsky-stained cytology slides (RSC) and to the matched cell blocks (CB) in 53 canine and feline lymphomas and 4 lymphocyte-rich (chylous) effusions.

Table S3: Matrix comparing the positivity scores of immunolabeling of CD3 in previous-stained cytology (RSC) and in cell blocks, as assessed by a board-certified pathologist. Score 0: no positive cells; score 1: ≤25%; score 2: >25 and ≤50%; score 3: >50 and ≤75%; score 4> 75% of positive cells.

**RSC**	**CB**				
	**0**	**1**	**2**	**3**	**4**
**0**	10	8	1	3	8
**1**	3	8	1	0	2
**2**	1	0	0	1	0
**3**	0	0	0	0	3
**4**	0	0	0	0	4

Table S4: Matrix comparing the intensity of immunolabeling of CD3 in previous-stained cytology (RSC) and in cell blocks (CB), as assessed by a board-certified pathologist. 0: no positive cell; level 1: weak staining; level 2: moderate staining; level 3: intense staining, as compared to controls.

**RSC**	**CB**			
	**0**	**1**	**2**	**3**
**0**	0	1	6	7
**1**	0	1	8	10
**2**	0	0	0	9
**3**	0	2	0	7

Table S5: Matrix comparing the background staining after the immunolabeling of CD3 in previous-stained cytology (RSC) and in cell blocks (CB), as assessed by a board-certified pathologist. 0: no background staining; 1: weak; 2: moderate; 3: intense background staining.

**RSC**	**CB**			
	**0**	**1**	**2**	**3**
**0**	26	4	0	0
**1**	10	3	0	0
**2**	5	3	0	0
**3**	1	0	0	0

Table S6: Matrix comparing the presence non-specific staining after the immunolabeling of CD3 in previous-stained cytology (RSC) and in cell blocks (CB), as assessed by a board-certified pathologist. 0: non-specific staining absent; 1: non-specific staining present.

**RSC**	**CB**	
	**0**	**1**
**0**	32	2
**1**	15	3

Supplemental Tables S7–S10: Contingency tables comparing the results of immunolabelling for PAX5 applied to previous Romanowsky-stained cytology slides (RSC) and in matched cell blocks (CB) in 53 canine and feline lymphomas and 4 lymphocyte-rich (chylous) effusions.

Table S7: Matrix comparing the positivity scores of immunolabeling of PAX5 in previous-stained cytology (RSC) and in cell blocks (CB), as assessed by a board-certified pathologist. Score 0: no positive cells; score 1: ≤25%; score 2: >25 and ≤50%; score 3: >50 and ≤75%; score 4> 75% of positive cells.

**RSC**	**CB**				
	**0**	**1**	**2**	**3**	**4**
**0**	10	7	0	0	2
**1**	4	13	1	0	1
**2**	0	0	0	0	0
**3**	0	1	1	1	1
**4**	1	0	1	3	9

Table S8: Matrix comparing the intensity of immunolabeling of PAX5 in previous-stained cytology (RSC) and in cell blocks (CB), as assessed by a board-certified pathologist. 0: no positive cell; level 1: weak staining; level 2: moderate staining; level 3: intense staining, as compared to controls.

**RSC**	**CB**			
	**0**	**1**	**2**	**3**
**0**	2	0	3	5
**1**	1	0	2	6
**2**	2	0	2	18
**3**	1	1	0	11

Table S9: Matrix comparing the background staining after the immunolabeling of PAX5 in previous-stained cytology (RSC) and in cell blocks (CB), as assessed by a board-certified pathologist. Score 0: no background staining; 1: weak; 2: moderate background (intense background level 3 was not observed).

**RSC**	**CB**		
	**0**	**1**	**2**
**0**	30	6	0
**1**	11	0	1
**2**	2	3	2

Table S10: Matrix comparing the presence non-specific staining after the immunolabeling of PAX5 in previous-stained cytology (RSC) and in cell blocks (CB), as assessed by a board-certified pathologist. 0: non-specific staining absent; 1: non-specific staining present.

**RSC**	**CB**	
	**0**	**1**
**0**	38	6
**1**	7	4
